# Trends and Gaps in National Blood Transfusion Services — 14 Sub-Saharan African Countries, 2014–2016

**DOI:** 10.15585/mmwr.mm6750a4

**Published:** 2018-12-21

**Authors:** Udhayashankar Kanagasabai, Michelle S. Chevalier, Bakary Drammeh, Fatima D. Mili, Michael L. Qualls, Naomi Bock, Irene Benech, Lisa J. Nelson, George Alemnji, D. Heather Watts, Daniel Kimani, Dejana Selenic

**Affiliations:** ^1^Epidemic Intelligence Service, CDC; ^2^Division of Global HIV/AIDS and Tuberculosis, Center for Global Health, CDC; ^3^CDC Uganda; ^4^Office of the Global AIDS coordinator and Health Diplomacy; ^5^CDC Kenya.

## Abstract

Ensuring availability of safe blood products through recruitment of voluntary, nonremunerated, blood donors (VNRDs) and prevention of transfusion-transmissible infections (TTIs), including human immunodeficiency virus (HIV), hepatitis B virus (HBV), hepatitis C virus (HCV), and syphilis, is important for public health (*1*,*2*). During 2004–2016, the U.S. President’s Emergency Plan for AIDS Relief (PEPFAR) provided approximately $468 million in financial support and technical assistance* to 14 sub-Saharan African countries^†^ with high HIV prevalence to strengthen national blood transfusion services (NBTSs)^§^ and improve blood safety and availability. CDC analyzed these countries’ 2014–2016 blood safety surveillance data to update previous reports (*1*,*2*) and summarize achievements and programmatic gaps as some NBTSs begin to transition funding and technical support from PEPFAR to local ministries of health (MOHs) (*2*,*3*). Despite a 60% increase in blood supply since 2004 and steady declines in HIV prevalence (to <1% among blood donors in seven of the 14 countries), HIV prevalence among blood donors still remains higher than that recommended by the World Health Organization (WHO) (*4*). PEPFAR support has contributed to significant reductions in HIV prevalence among blood donors in the majority of PEPFAR-supported countries, and linking donors who screen HIV-positive to confirmatory testing and indicated treatment, as well as further reducing TTIs, remains a public health priority (*5*).

In 2016, WHO Global Status Report on Blood Safety and Availability^¶^ reported that 5.6 million units of blood (4% of the global supply) were collected in Africa; 38 African countries collected <10 whole-blood donations per 1,000 population, the WHO-recommended target (*1*). To meet demand, countries often rely on family or replacement donors who donate blood for a family member or friend; however, such donations carry a higher risk for TTIs (*6*). Since 2004, PEPFAR support has helped establish national blood policies, improved blood donor screening, increased recruitment and reliance on VNRDs for national supplies, and strengthened laboratory infrastructure, accreditation, information systems, and continuous quality improvement programs (*4*).

During 2014–2016, NBTSs in the 14 PEPFAR-supported sub-Saharan African countries used a standardized data collection tool to report the total number of blood units collected; the percentage of donated units that screened positive for HIV and other TTIs; the percentage of screen-positive donors who were notified of their result; and the status of financial support transition from PEPFAR to MOHs. MOH funding to support blood safety activities at the local NBTS was self-reported to the PEPFAR and CDC-supported WHO Global Database on Blood Safety. The numbers of whole blood units collected per 1,000 population per year were calculated using national census estimates or United Nations population projections.**

During 2004–2016, overall total annual blood collections in PEPFAR-supported countries increased 60%, from 1,469,561 units in 2004 to 2,352,905 units in 2016, although collection rates remain below WHO recommendations (*1*) in all countries except South Africa and Swaziland (Table 1). From 2014 to 2016, the number of units collected per 1,000 population decreased in five countries (Kenya, Lesotho, Nigeria, Swaziland, and Zambia); however, during this period, eight countries reported collecting 100% of their national blood supply from VNRDs. The largest increase in VNRD donations (40%) occurred in Ethiopia (from 70% in 2014 to 98% in 2016); however, declines in VNRD donations in Lesotho (18%, from 96% to 79%) and Tanzania (11%, from 89% to 79%) also occurred.

**TABLE 1:** Number of blood units collected by U.S. President’s Emergency Plan for AIDS Relief (PEPFAR)–supported blood transfusion services, number of blood units from voluntary nonremunerated donors (VNRDs), and blood units collected per 1,000 population, by country — 14 PEPFAR-supported countries, 2004 and 2014–2016

Country	2004			2014			2015			2016		
	No. collected	% VNRD	No. per 1,000 population	No. collected	% VNRD	No. per 1,000 population	No. collected	% VNRD	No. per 1,000 population	No. collected	% VNRD	No. per 1,000 population
Côte d'Ivoire	77,972	100	3.4	143,691	100	6.3	155,534	100	6.8	168,025	100	7.4
Ethiopia	43,247	59	0.4	87,685	70	0.8	140,061	97	1.4	173,923	98	1.7
Ghana	165,426	41	6.0	150,322	30	5.4	155,250	34	5.6	160,624	36	5.8
Kenya	18,440	100	0.4	183,475	100	3.9	155,081	100	3.3	167,100	100	3.6
Lesotho	3,000	95	1.4	8,373	96	3.9	7,879	97	3.7	5,008	79	2.3
Mozambique*	67,105	58	3.4	121,091	39	4.3	126,068	42	4.5	131,231	45	4.6
Nigeria^†^	1,266	100	<0.1	48,908	91	0.2	66,614	82	0.3	51,329	84	0.2
Rwanda	28,777	100	2.4	42,789	100	3.6	53,436	100	4.6	61,768	100	5.3
South Africa	709,324	100	13.0	803,818	100	14.7	828,689	100	15.2	810,895	100	14.8
Swaziland	7,060	100	5.4	14,727	100	11.3	13,752	100	10.5	13,687	100	10.5
Tanzania^§^	129,404	66	2.4	128,915	89	2.4	67,980	49	1.2	196,735	79	3.6
Uganda	112,250	100	2.8	212,939	100	5.4	230,995	100	5.9	243,335	100	6.2
Zambia	38,477	71	2.3	109,269	100	6.7	100,110	100	6.1	104,355	100	6.4
Zimbabwe	67,813	100	4.3	58,603	100	3.7	59,767	100	3.8	64,890	100	4.1
**Total**	**1,469,561**	**—**	**2.2**	**2,114,605**	**—**	**3.4**	**2,161,216**	**—**	**3.6**	**2,352,905**	**—**	**3.8**

**Source:** 2004, 2014–2016 population data from the Joint United Nations Programme on HIV and AIDS. http://aidsinfo.unaids.org/.

**Abbreviations:** AIDS = acquired immunodeficiency syndrome; HIV = human immunodeficiency virus.

* 2004 data for Mozambique from https://www.cdc.gov/mmwr/volumes/65/wr/mm6505a4.htm.

^†^ Nigeria and Tanzania did not have data for 2004; therefore, data for 2003 and 2005 were used.

In all 14 countries, most blood donors were men (65% in 2014 and 86% in 2016); however, from 2014 to 2016, the number of female blood donors aged 20–24 years increased approximately thirtyfold, from 4,424 in 2014 to 146,571 in 2016. The largest increase in male donors (201%) occurred among persons aged 30–34 years, from 45,725 in 2014 to 137,596 in 2016.

During 2014–2016, the prevalence of whole blood units screening positive for HIV declined in 10 countries (range = 0.1–1.2 percentage-point declines) but increased in Nigeria (by 0.1 percentage point), Rwanda (0.1) and Swaziland (1.2) (Table 2). The HIV screening prevalence among donated units in seven countries remains higher than the WHO target of <1% (*4*). During 2014–2016, in nine countries with information on informing donors of HIV screening results, only 18.0% (2,971 of 16,539 [2014]) to 27.6% (3,660 of 13,269 [2016]) of donors who screened HIV-positive were notified of their results (Figure). During this period, the total number of deferrals remained steady (>250,000 units); however, deferrals attributable to high-risk behavior declined from 2014 to 2015.

**TABLE 2:** Population prevalence of human immunodeficiency virus (HIV) infection among persons aged 15–49 years in the general population, percentage of collected blood units reactive for HIV, and percentage of collected blood units reactive for three transfusion-transmissible infections (TTIs) (hepatitis B virus [HBV], hepatitis C virus [HCV], and syphilis), by country — 14 U.S. President’s Emergency Plan for AIDS Relief–supported countries, 2014–2016*

Country	HIV population prevalence (%)			Prevalence (%) of TTIs in collected blood units								
				HIV			Other TTIs			All TTIs		
							HBV, HCV, and syphilis			HIV, HBV, HCV, and syphilis		
	2014	2015	2016	2014	2015	2016	2014	2015	2016	2014	2015	2016
Côte d'Ivoire	3.0	2.8	2.7	0.3	0.04	0.2	8.6	9.0	8.9	9.0	9.0	9.1
Ethiopia	1.1	1.1	0.9	2.1	1.2	1.1	4.4	4.6	4.2	5.2	5.1	4.5
Ghana	1.7	1.6	1.6	0.7	0.5	0.3	9.7	7.1	11.6	11.8	8.3	12.7
Kenya	5.7	5.6	5.4	0.6	0.8	0.6	2.8	4.3	2.5	3.5	5.2	3.2
Lesotho	24.7	24.9	25	2.6	2.4	2.5	3.6	3.8	5.0	6.2	6.2	7.6
Mozambique	13.0	12.7	12.3	5.2	4.8	4.0	8.2	8.8	6.9	13.4	13.6	11.0
Nigeria	3.1	3.0	2.9	1.4	1.4	1.5	11.3	11.7	13.1	12.9	13.2	14.6
Rwanda	3.2	3.2	3.1	0.1	0.1	0.2	2.6	2.7	3.4	2.8	2.9	3.6
South Africa	18.8	18.9	18.9	0.2	0.2	0.1	0.3	0.3	0.5	0.5	0.5	0.7
Swaziland	27.6	27.5	27.2	0.7	1.5	1.9	1.6	3.0	5.6	2.4	4.6	7.6
Tanzania	6.9	6.7	6.5	1.4	1.5	1.3	7.7	14.3	7.6	9.2	10.8	8.9
Uganda	5.0	4.8	4.7	0.9	0.6	0.6	3.4	4.2	3.8	4.3	4.8	4.4
Zambia	12.7	12.6	12.4	3.4	2.9	2.9	8.1	7.1	7.0	11.6	10.1	10.0
Zimbabwe	14.3	13.9	13.5	0.5	0.4	0.4	0.6	0.4	0.4	1.1	0.8	0.8

**Source:** 2014–2016 from United Nations Development Program population estimates. http://hdr.undp.org/en/data#.

**Abbreviation:** AIDS = acquired immunodeficiency syndrome.

* Self-reported data for 2014, 2015, and 2016.

**FIGURE:**
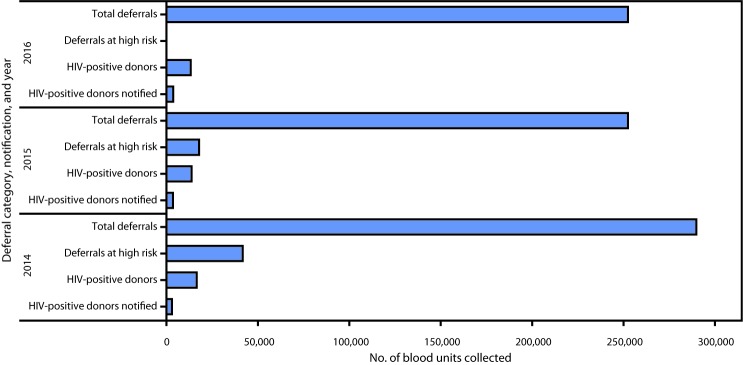
Total number* of blood units collected for all deferrals,^†^ deferrals at high risk,**^§^****^,^****^¶^** human immunodeficiency virus (HIV)–positive donors, and HIV-positive donors notified of their HIV status, by year — nine U.S. President’s Emergency Plan for AIDS Relief–supported countries,** 2014–2016^††^ **Abbreviation:** AIDS = acquired immunodeficiency syndrome. * Total number of blood units collected: 1,583,617 in 2014; 1,590,104 in 2015; and 1,771,798 in 2016. **^†^** Deferrals are defined as donors who do not meet donor selection criteria after administration of a risk assessment questionnaire. ^§^ Deferrals at high risk, classified based on seven categories of behavior; data for number of deferrals at high risk from Global Database for Blood Safety. ^¶^ Percentage of deferrals at high risk from total blood units collected: 2014, 14%; 2015, 7%; and 2016, data not available. ** Ethiopia, Kenya, Nigeria, Rwanda, South Africa, Swaziland, Tanzania, Uganda, and Zambia. ^††^ Number of deferrals at high risk for 2016 was not available.

From 2014 to 2016, the prevalence of HBV, HCV, and syphilis reactivity in donated blood units decreased in six countries; decreases ranged from 0.1 percentage point (Tanzania) to 1.3 percentage points (Mozambique) (Table 2). The prevalence of TTIs in donated units increased in seven countries (Côte d’Ivoire, Ghana, Lesotho, Nigeria, Rwanda, South Africa, Swaziland, and Uganda) (Table 2). In 2016, the percentage of donated blood units that screened positive for all TTIs ranged from 0.7 (South Africa) to 14.6 (Nigeria).

As support for local blood safety programs transitioned to MOHs from PEPFAR, MOHs in Ethiopia, Swaziland, and Tanzania completely absorbed the cost of collecting and testing blood in 2016. Nine of 12 countries with available data report ≥50% of MOH support to the NBTS (Supplementary Table, https://stacks.cdc.gov/view/cdc/61188).

## Discussion

Sub-Saharan African countries have improved access to safe and adequate blood supplies, but continued commitment and funding are required to maintain gains and achieve WHO targets. Although the number of blood units collected has increased since 2004, whole blood collections remain insufficient to meet national demand: 12 of 14 evaluated countries do not meet the WHO-recommended target (*1*). This shortfall especially affects women with pregnancy-related complications, trauma victims, and children with severe life-threatening malaria-related anemia (*7*).

Although most of the 14 PEPFAR-supported countries reported decreases in the percentage of collected blood units that screened positive for HIV since 2004, percentages remain significantly higher than the 0.003% reported by high-income countries (*1*). Seven countries have HIV screen-positive rates that exceed the WHO recommended target of <1.0%. Although HIV prevalence rates among blood donors have decreased, prevalences of other TTIs such as HBV, HCV, and syphilis increased in seven countries. To reduce the risk for TTIs in sub-Saharan Africa when PEPFAR support ends, MOHs can participate in cross-sector collaborations to implement blood bank quality and safety accreditation standards through the African Society for Blood Transfusion (AfSBT)^††^ or other international accrediting bodies and implement PEPFAR-supported blood safety information systems. Recent data indicate that 50% of PEPFAR-supported countries still do not have a computerized information system for blood donor tracking and TTI testing. Since 2016, blood safety information systems have been implemented in three countries, with another two planned by 2019. To date, only four NBTSs in sub-Saharan Africa (Namibia [accredited in 2012], South Africa, Rwanda, and Tanzania) have achieved accreditation by an external body. Seven countries are currently in various stages of the accreditation process through AfSBT. Global CDC blood safety targets are that 50% of NBTS sites reach at least the first of three accreditation steps under AfSBT during the next 2–3 years.

As countries move toward the United Nations 95–95–95 targets (95% of HIV infection diagnosed, 95% of infected persons receiving antiretroviral therapy [ART], and 95% of those on ART achieving viral suppression) for achieving epidemic control, increasing outreach to priority populations for testing and preventive services become increasingly important (*8*). Currently, no systems exist within these NBTSs to link persons determined to be ineligible for donation through behavioral risk screening to HIV testing and preventive services.

During 2014–2016, four NBTSs transitioned from PEPFAR to full MOH funding. An additional five countries received ≥50% of their funding from MOHs; two countries reported a decrease in MOH funding. As PEPFAR transitions occur, countries should consider prioritization of funding to their NBTS to sustain the gains achieved (*9*).

The findings of this report are subject to at least four limitations. First, blood unit collections described in this report only represent units collected by the NBTS, and do not account for units collected in the private sector or by nonnational blood transfusion services. Second, variations in testing capacity and assays used for laboratory screening (most NBTSs lack HBV and HCV confirmatory testing) might result in over- or underestimation of TTI prevalence rates among blood donors. Third, lack of information systems to link donors who screen HIV-positive to treatment services might result in inaccurate estimations of the number of donors who are notified about their status. Finally, self-reported data from countries might result in inaccurate estimations.

A decade of PEPFAR support to NBTSs in 14 countries has led to increases in blood collections, fewer donors screening HIV-positive, and transition of support from PEPFAR to MOHs. However, gaps in linking deferred donors at high risk to HIV testing and prevention services, and in notifying HIV-positive donors of their status and linking them to confirmatory testing, care, and treatment underscore the need for enhanced focus on epidemic control, as well as innovative strategies to address donors who test positive for other TTIs. Ending reliance on unsafe blood donors requires continued investment in laboratory quality improvement, including increased engagement in external proficiency testing and increased use of highly sensitive assays at the NBTS and non-NBTS testing sites. Continued improvement of blood safety programs in sub-Saharan Africa will require sustained investments in continuous quality improvement, NBTS accreditation under AfSBT, linkage of deferred donors who report high risk behaviors and those who screen HIV-positive to HIV testing services and treatment, and stronger blood safety information systems. Strengthening health systems and developing local policy and sustainable financial resources are all important components to consider to ensure the future viability of blood safety programs.

**Summary**

**What is already known on this topic?**

Since 2004, the U.S. President’s Emergency Plan for AIDS Relief has improved blood availability and safety in 14 sub-Saharan African countries; however, the risk for human immunodeficiency virus (HIV) transmission via transfusion remains unacceptably high.

**What is added by this report?**

During 2014–2016, blood collections increased and donor HIV prevalence decreased in seven of the 14 countries, but systems to link HIV-positive and donors at high risk to testing and treatment are inadequate.

**What are the implications for public health practice?**

Sustained investments by ministries of health in continuous quality improvement, national blood transfusion services accreditation, linkage of HIV-positive and donors at risk to testing, care, and treatment, and blood safety information systems remain important components to ensure the viability of blood safety programs.

## Footnotes

* Blood Safety programs are funded through the Human Movement Blood Laboratories budget code.

† Côte d’Ivoire, Ethiopia, Ghana, Kenya, Lesotho, Mozambique, Nigeria, Rwanda, South Africa, Swaziland, Tanzania, Uganda, Zambia, and Zimbabwe. A full list of countries receiving PEPFAR support is available at https://www.pepfar.gov.

§ National blood transfusion services refers to those government or nongovernmental organizations with a legal mandate to collect, test, process, and distribute blood and blood components within a given country, or the legal authority to oversee or regulate the collection, testing, processing, and distribution of blood and blood components by other entities within that country.

¶ https://www.who.int/bloodsafety/global_database/en/.

** Africa Society for Blood Transfusion accreditation lasts for 2 years; Namibia was accredited in 2012.

†† https://afsbt.org/.
